# Men’s Voices on Long-Acting Pre-Exposure Prophylaxis Delivery Modalities: Acceptability and Preferences Among Cisgender Men and Men Who Have Sex With Men in South Africa

**DOI:** 10.1097/QAI.0000000000003638

**Published:** 2025-05-01

**Authors:** Millicent Atujuna, Alinda M. Nyamaizi, Zoe Duby, Alexandra Minnis, Miranda Diaz, Thesla Palanee-Phillips, Siyanda Tenza, Krishnaveni Reddy, Nqaba Nkomana, Linda-Gail Bekker, Elizabeth T. Montgomery

**Affiliations:** a Desmond Tutu HIV Centre, Faculty of Health Sciences, University of Cape Town, Cape Town, South Africa; b Women’s Global Health Imperative at RTI International, Research Triangle Park, NC; c Department of Maternal and Child Health, University of North Carolina at Chapel Hill, NC; d Division of Social and Behavioural Sciences, School of Public Health and Family Medicine, University of Cape Town, Cape Town, South Africa; e Wits RHI, School of Public Health, Faculty of Health Sciences, University of the Witwatersrand, Johannesburg, South Africa; f Department of Epidemiology, School of Public Health, University of Washington, Seattle, WA.; g University of California, San Francisco, USA

**Keywords:** LA PrEP, South Africa, MSM, qualitative research, HIV, heterosexual men

## Abstract

**Introduction:**

In sub-Saharan Africa, cisgender men—in particular men who have sex with women (MSW) and, to a lesser degree, men who have sex with men (MSM)—are often under-represented in HIV prevention research, despite their own HIV risk and role in transmission cycles. As HIV prevention research on long-acting pre-exposure prophylaxis (LA PrEP) options expands in sub-Saharan Africa, it is essential to engage these populations to ensure their acceptability. We investigated perceptions of implants and intramuscular injectables as LA PrEP delivery among MSW and MSM.

**Methods:**

In-depth interviews were conducted between October 2020 and March 2021 with 40 MSW (n = 20) and MSM (n = 20), aged 18–35 years, self-reported as HIV negative, sexually active, and residing in resource-restricted communities in Cape Town and Johannesburg, South Africa. We explored factors influencing LA PrEP attitudes. Data analysis followed a thematic framework approach.

**Results:**

MSW and MSM found LA PrEP administration modes more acceptable than daily oral PrEP because they offered longer lasting protection while reducing frequent clinic visits for refills. MSW voiced hesitancy around the use of “foreign products,” fearing infertility and congenital disabilities in their future children. Both subgroups acknowledged the convenience of implants with long-dosing duration, but injections were deemed to be more discrete and familiar. Both groups described implant use as potentially stigmatizing, with a greater chance of causing tissue scarring from insertion and removal procedures.

**Conclusions:**

Evidence relating to men’s engagement in HIV prevention and acceptable modalities of HIV prevention is limited. We found that both groups were enthusiastic about LA PrEP, informing the development of our subsequent clinical study to provide further insight into using placebo versions of LA PrEP and future implementation of LA PrEP options.

## INTRODUCTION

Cisgender men who have sex with women (MSW) and men who have sex with men (MSM) are often under-represented in HIV prevention research and HIV programming in sub-Saharan Africa (SSA)—albeit for different reasons—despite their undeniable role in HIV transmission. For MSW, their lower HIV incidence estimates are often cited as the main reason for this gap, yet men have been shown to be less likely to know their HIV status, engage in prompt HIV care, sustain care, and maintain a suppressed viral load, when compared with women.^1–4^ Consequently, MSW have a higher HIV mortality rate than women in SSA.^5–7^ By contrast, MSM, who comprise a diverse population with a variety of gender identities, have been defined as a priority population in the global HIV epidemic response, given their 25 times higher risk for HIV acquisition than MSW.^6,8,9^ In the SSA region, however, rampant homophobia, criminalization, and marginalization of MSM continue to threaten how MSM engage in HIV intervention programs.^5,10,11^

The current landscape for HIV prevention consists primarily of male condoms and oral pre-exposure prophylaxis (PrEP), both highly effective tools when used as prescribed and even more so when combined.^3^ However, both these products are associated with widespread adherence challenges. Numerous factors motivate men to opt for condomless sex, including enhanced sexual pleasure, increased intimacy, and a complex interplay of gendered sexual norms, masculinities, and dyadic power dynamics common in many African and South African contexts.^12^ The introduction of oral PrEP was hailed a solution to some of the aforementioned challenges of condom use, affording users increased intimacy and sexual pleasure, and reduced anxiety regarding HIV transmission during intercourse.^13^ However, despite its demonstrated efficacy and reported benefits, uptake of and adherence to oral PrEP, particularly among MSM, have been discouraging.^13,14^ Men’s poor adherence to oral PrEP, like for women,^15–18^ is largely attributable to the burden of taking a pill daily, fears related to side effects, and PrEP-related stigma associated with perceptions of PrEP being treatment rather than prevention, anticipated stigma because of fear of being labeled as HIV positive for taking PrEP, and assumptions of PrEP users as promiscuous/“high risk.”^14^

By contrast, long-acting (LA) implants and injectables are comparatively discrete and less burdensome to end users than oral PrEP.^19^ Implants, although still in the early stages of development, demonstrate promising advantages over LA injectable formulations, including the capacity to release therapeutic drug levels in excess of 12 months, delivery at a constant flat rate with a limited period of subtherapeutic tailing, removability in case of adverse reactions, and compatibility with highly soluble antiretroviral agents.^19,20^ Investigating the acceptability of LA PrEP delivery platforms among men is crucial for successful product design. Injectable cabotegravir for LA PrEP showed high acceptability among HIV-uninfected men and women in the recent HTPN-077 study.^20^ In the HPTN-083 study, injectable cabotegravir(administered through bimonthly intramuscular gluteal injections) was evaluated in HIV-uninfected cisgender MSM and transgender women across 7 countries, including South Africa and found to be superior to daily oral PrEP in preventing HIV infection and in the absence of safety concerns.^21^

However, there is limited literature on implant acceptability for men.^22–26^ Because HIV prevention research on LA PrEP options expands in SSA, engaging all men, regardless of their sexual identities, is essential to ensure their acceptance of this modality. Previous formative research in Cape Town, South Africa, showed that young men were interested in using injectable and implantable products for HIV prevention, however, these indicated preferences were based on hypothetical scenarios only in a discrete choice experiment.^27^ Previous studies with placebo products among women have demonstrated that acceptability and preference for LA PrEP delivery platforms changed pre- and postuse of the products under study, highlighting the need for a similar investigation with men.^28^ To address this gap, the South African Male-User Research about Acceptability of Implants and Injections (SAMURAI) for HIV prevention study formative work, presented here, investigated perceptions of implants and injectables as LA PrEP delivery platforms among MSM and MSW in South Africa to inform the recruitment procedures of the clinical component of the study and understand men’s acceptability of these LA PrEP.

## METHODS

### Study Design and Setting

The formative research component of the SAMURAI study consisted of a series of in-depth interviews (IDIs) designed to identify perceptions of implants and injectables as delivery methods for LA PrEP, conducted between October 2020 and March 2021. Participants were primarily recruited from 2 communities in Cape Town and Johannesburg, South Africa. In Cape Town, the study took place in the community of Phillippi and the surrounding townships including Nyanga, located in the Cape Town metropolitan area. These areas are densely populated, have a high HIV burden, and are poorly resourced. In Johannesburg, this study took place within Hillbrow, an inner city residential neighborhood that serves as a port of entry for migrants and immigrants from townships, rural areas, and other parts of Africa, and as such nurtures a highly transient and at-risk population.

### Participants

To ensure a diverse range of perspectives on knowledge, attitude, perceptions, barriers/facilitators to LA delivery, and engagement of clinical care, participants were recruited from previous HIV prevention studies (such as oral PrEP clinical trials where PrEP was dispensed monthly to assess adherence efficacy) and through in-person, online community venues, and peer recruitment. Participants included heterosexual men (n = 20) and MSM (n = 20) aged 18–35 years. In this study, the term “heterosexual men” referred to cisgender men who have sex only with women (MSW), while MSM described cisgender men who have sex with men. Eligible participants within the age and subgroup parameters were self-reported as being HIV negative and currently sexually active. Potential eligible participants were prescreened using a checklist completed face to face. During this process, participants who were in a private setting informed that this study aimed to obtain voices of participants who identified as “straight men” or “MSM.” They were then asked whether they identified as either. Based on their response, we were able to determine their sexual orientation and allocation to a participant group. Participants provided written informed consent conducted in private, before any study procedures.

### Data Collection

**FIGURE 1.**
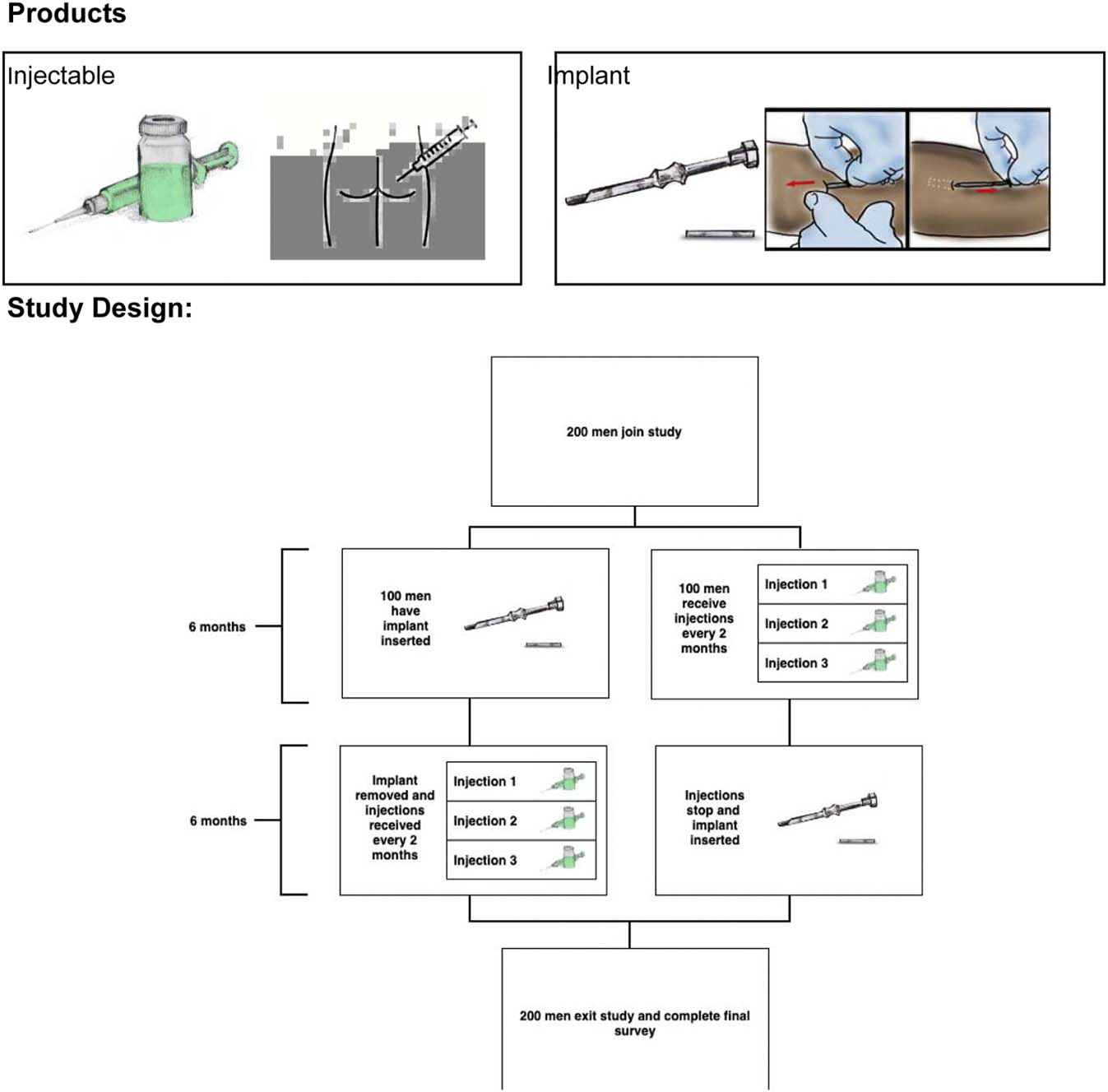
South African Male User Research on Acceptability of Implants and Injections (SAMURAI) visual tool.

A short questionnaire was administered to capture basic demographic information and health knowledge. An IDI (~1 hour long) was conducted to explore participants’ attitudes toward and preferences for implants versus injectable formulations of LA PrEP. To facilitate understanding of these PrEP methods, the team provided a concise standard explanation of each product, emphasizing that these products are designed for HIV prevention and are either in development or undergoing research trials. The explanation included details on dosing and routes of administration. A visual tool (Fig. 1) was used to enhance comprehension of the 2 LA PrEP options. Other areas of inquiry in the IDI guide included factors influencing engagement with care and product use, including gender norms and stigma, and perceived behavioral control.

Interviewers followed piloted semistructured interview guides, developed through literature review and informed by socioecological and acceptability frameworks.^29,30^ This framing positioned men’s behaviors and preferences in a broader context, informed by interpersonal, organizational (eg, health system), and structural (eg, stigma, homophobia, gender norms) factors. We explicitly investigated these contextual influences and their impact on shaping user experience to identify user preferences regarding HIV prevention products and how product characteristics influence product buy-in.

### Data Analyses

Interviews were conducted in-person in the participants’ preferred language (English or IsiZulu in Johannesburg and Xhosa in Cape Town). Interviews were audio-recorded, transcribed, and translated into English where necessary. Textual data coding was the primary qualitative analytical approach to summarize, extract meaning, and condense the data from the full IDI transcripts, using Dedoose qualitative software. First, we used descriptive coding for key themes and topics, using a preliminary codebook. Additional codes were identified through an iterative process of reading the textual data to identify emergent themes, and the codebook was modified accordingly. Pattern codes, which achieved a greater level of abstraction, were used to link themes and topics together to explore the relationship between the various constructs described in the theoretical framework. Beyond allocating sections of text under thematic areas, analysis involved the use of analytic summary memos, in which analysts articulated interpretations of the data in a concise format, helping to create a deeper level of interpretation and narrative.^31^ The findings and interpretations of the data were critically discussed by the analysts and investigators until group consensus was reached on the dominant themes and meanings.

### Ethical Considerations

Ethics approval was granted by the University of Cape Town’s Human Research Ethics Committee(HREC (Ref#: 277/2020)), Wits University’s Human Research Ethics Committee (Wits HREC Ref#: 200508), and the sponsoring site in the United States (Ref#: 00000925). Participants provided written informed consent before data collection, and all personal identifying information was kept confidential. To ensure participant confidentiality, all data collected were stored in a secure location, accessible only by authorized members of the research team.

## RESULTS

A total of 40 participants completed IDIs as given in Table 1. The median age of the participants was 23.5 years. Among the men interviewed, 55% belonged to the Xhosa ethnic group and 52.5% reported primarily speaking IsiXhosa at home. Half of the sample had completed secondary school, while 32.5% were currently enrolled in school. Regarding sexual orientation, 42.6% identified as gay, 47.5% as straight, and 10% as bisexual. In our sample, 8 MSM were trial naive and 12 were trial experienced. Among MSW, 19 were trial naive and 1 was trial experienced. In addition, 80% of the participants reported having a primary partner, 47.5% used a condom during their last sexual encounter, and 75% reported not having a source of income.

### Results Overview

In exploring men’s views about LA PrEP, we identified 2 themes: (1) perceived benefits and concerns about LA PrEP and (2) preference for treatment modality (ie, injection versus implant).

### Theme 1: Perceived Benefits and Concerns About LA PrEP

#### Reducing the Burden of Daily Pill Intake and Frequent Clinic Visits

Both MSW and MSM indicated that the concept of an LA PrEP option, whether delivered through injections or implants (once available), was acceptable and more appealing than daily oral PrEP because they offered longer lasting protection while reducing the burden related to frequent clinic visits for refills. In their descriptions, men seemed to immediately foresee how these products were better alternatives to daily oral PrEP. They expressed the fear of being stigmatized as living with HIV while on oral PrEP, and having to frequently stand in long queues for PrEP refills. Moreover, men viewed frequent visits to the clinic as costly. For these men, long-acting products have the potential to overcome oral PrEP-associated challenges they had experienced as exemplified by this participant’s response:

“It (the implant) also could be helpful because some of us (men) don’t like clinic, a person prefers to go once in a while so like at least when he knows you have your implant there’s nothing that makes him go to clinic every time because like he knows that he will be going to check every six months still [to check whether he is still] on the right track then if it’s still active... even to go for HIV testing once in three months we don’t do that... I must come to clinic once after a while because like other people you will find it difficult to get off days... (men) will use implant because others they are scared of clinic because the minute you mention clinic it means you are going to eat pills for HIV and AIDs. A person will enjoy the fact that it’s a year there nothing will make him to go there always of which that way its manageable and also at work they will not be nervous you are no longer going to clinic always like every month or every two months...” (IDI#2106, MSM, Cape Town)

For the MSM group, clinic attendance was associated with being identified (and then potentially stigmatized) as “gay” by other community members. This fear was well articulated, and they hoped this would be minimized by the use of LA PrEP as illustrated in the below statement:

“...another thing with men is that men do not like going to the clinic so with PrEP every two months you have to go for your supply more especially with men’s clinic because everyone thinks that if you go there, you are gay because they promote a lot of LGBTQI friendly services, so even if you are straight when you go they think you are gay... in the community when you go to the men’s clinic... they know that only gay people go there so they assume that you are gay... with implant you always renew it maybe after a year.” (IDI#1108, MSM, Johannesburg)

#### Provides Protection That Is Less Prone to Interruption Along With Enhanced Sexual Pleasure

Many of the MSW and MSM described these new products as having the potential to overcome various challenges relating to inconsistent condom use while offering a more acceptable tool to reduce the risk of acquiring HIV. Although they described being aware that condoms are protective against other STIs and unwanted pregnancies with their partners, these participants explained that consistent condom use was still low. They explained that their preference for condomless sex was for pleasure and intimacy although sometimes this was compounded by other risk factors occurring in tandem, such as alcohol use. These men felt that using LA PrEP, much like oral PrEP, provides a better and more feasible option that would see them protected from HIV, while experiencing maximum sexual pleasure, without a condom, as described below:

“I think it (new products-LA PrEP) will help, most people are sick and we don’t know why they get sick. We saw people dying in front of us...people will hide the cause of death and they say he died because of asthma instead of HIV. I think this will help us in fact it will help me, I am always the one who doesn’t use condoms, I don’t even like it so it (new products) will help me in many things...” (IDI#2101, MSM, Cape Town)

#### Product Mistrust

However, some men voiced their hesitancy around the use of “foreign products” because they expressed their mistrust toward these products in general. “Foreign” for potentially 2 reasons: because they are not innately part of one’s natural biology and especially they are manufactured and introduced by scientists outside of South Africa. Their distrust was, however, not just regarding where products were manufactured but also related to the products’ chemical composition and formulations:

“I will want to know where they (products) come from how are they manufactured, what ingredients they use to make this HIV prevention product... It is important because you know some of the goods that are transported from one country to another can be swapped so I think that is my concern. It is risky to use the products that you don’t know where they come from, what if on their way they switch the products to something else? ...(but) I want to try some new things that will prevent me from getting HIV that is why I am interested...” (IDI#1106, MSM, Johannesburg)

Most MSW also expressed concerns about potential intervention side effects such as infertility and possible birth defects in their future children.

“My concern is that it doesn’t kill my fertility...it must not disturb my ability to have children... Because I still want to have children...” (IDI#2010, MSW, Cape Town)

“Maybe if something happens with your sperm you know like maybe you can’t produce anymore maybe your kids come out... I don’t know, there are some deficiencies …” (IDI#1005, MSW, Johannesburg)

### Theme 2: Preference for Injectables Versus Implants

#### Men Appreciated the Convenience and Long Dosing Duration of Implants but Disliked the Potential Stigma and Higher Risk of Tissue Scarring

The convenience of implants with long dosing duration was acknowledged by both participant groups, but injections were deemed to be more discrete and familiar. Those who preferred the implant articulated it in a manner that categorized the implant as a product that does not require much attention or follow-up because “you just insert it and forget about it” (IDI#1105, MSM participant, Johannesburg), while knowing that “you are covered [protected] for 6 months” (IDI#1108, MSM, Johannesburg), which would boost sexual pleasure and confidence about one’s HIV prevention.

Some men were quick to express their reservations using an implant because they perceived the implant to be potentially stigmatizing, because it has a greater chance of causing tissue scarring from implant insertion and removal procedures, and is thus potentially less discrete. The stigma-related concerns related to implant insertion arose from 3 identifiable fears or perceptions: First, participants from both the MSW and MSM group articulated they felt uncomfortable about having implants in their bodies, because they feared being identified as having “foreign objects” in their bodies (IDI#2105, MSM, Cape Town). This is illustrated by the following statement: “They [men] will fear the implant because we are traditional people. When you tell them (that you had an implant) they will be like ‘hey you are carrying things now [having a physical object in the body]’.” (IDI#1007, MSW, Johannesburg).

Second, some men feared that having an implant could be associated or misconstrued as being seriously ill and that the purpose of the implant was a life-sustaining measure: “...I think the injection you can tell them that you took it, but the implant is like you are carrying something that is protecting you [keeping you alive] they will think that you are sick...” (IDI#1007, MSW, Johannesburg). Third, participants feared the stigma that emanated from the perceived notion that implants were women’s products, given the familiarity with the female contraceptive implant: “...they (men) will have that stigma that, ‘no, no this is woman’s stuff’...women do that, not men. They would make fun of, of you being a man and maybe you my brother, these are my friends they would be like, ‘man, you got an implant? No, my wife has that.’ You understand. They would make fun...definitely.” (IDI#1103, MSM, Johannesburg). Another participant described that, “They (men) will prefer injection because they will say implant it’s designed for girls.” (IDI#2109, MSM, Cape Town). In addition, some participants also raised concerns that the use of implants would inhibit their ability to conduct physical activity (eg, exercise).

#### Men Preferred the Discretion of Injections but Disliked the Need for Frequent Clinic Visits

In contrast, even though HIV prevention injections will require more frequent administration than the implants, injections’ discreteness was still favored. Participants explained that because the injection goes straight into the body (through intramuscular administration, ie, systemically distributed), and dissolves fast, it can be used without anyone else’s knowledge. They further expressed that once it is in the body, one does not have to worry about it, unlike the implant, which might be felt if someone touches the arm, or oral PrEP, which one has to remember to take every day. In addition, many participants expressed their preference for the injection because they felt that it was familiar, with many having received injections for other reasons throughout their lives:

“...the injection is cool for me... we black men we are more traditional... We have so many beliefs. So once you start telling us you want to put something that is going to remain inside of us [referring to an implant], it just becomes a no. So, injections are normal things that we know are used to injections we grew up seeing injections...” (IDI#2002, MSW, Cape Town).

Three participants from both the MSW and MSM groups, however, expressed their fear of potential pain resulting from injections administration. Others were concerned about forgetting repeat visits, losing interest, and their dislike toward the injection administration location—the bum—that they deemed to be private.

Despite the concerns raised above, participants from both MSM and MSW groups were optimistic about LA PrEP to the extent that they gave suggestions regarding what attribute they would want in LA PrEP. For example, they stated that LA PrEP should be as discrete as possible. It should be invisible, and impalpable under the skin, and must be inserted somewhere not noticeable and more hidden in the body (eg, under the arm, stomach, bum). For the injection, participants mentioned their preference for a self-injecting (at home) option. This choice was motivated by the desire to avoid discomfort associated with exposing themselves to health care providers, particularly when the provider was female. The participants were also open to the idea of having their loved ones administer the injection if they were unable to self-administer, indicating a level of comfort and trust in involving close individuals in their health care process. All participants preferred an option with a longer dosing duration beyond what is currently offered. Most men also preferred the concept of its administration to be on the arm rather than the buttocks, which again speaks of their desire for privacy. Other participants mentioned preferences for alternative administration methods, such as rectally inserted suppositories that could be used shortly before sexual activity, or those to be consumed orally (drinkable). Furthermore, participants discussed their discomfort with traditional clinics and expressed a desire for improved accessibility to LA PrEP. They cited the current widespread availability of condoms as an example and suggested that these new products should be accessible in various locations beyond clinics.

## DISCUSSION

Through the analysis of the IDIs administered to both MSM and MSW participants, 2 main themes emerged. First, cisgender men considered the concept of an LA PrEP option, irrespective of delivery method, acceptable and more appealing than daily oral PrEP, though not without some concerns. Probing of user experience and concerns regarding current PrEP options indicated that most participants found LA PrEP more feasible and amenable because of the increased perceived discretion this offered. When asked about delivery modality, participants favored the dosing duration and the convenience of less frequent clinic visits of the implants, while injections were preferred because of their discreteness, familiarity, and systemic administration. A second theme emerged regarding perceived external and internal stigma associated with HIV infection, health-seeking and long-acting products, and raised the possible need for alternative health care delivery systems for men. In addition, participants expressed hesitancy and mistrust toward “foreign” products, specifically concerns about their chemical composition and formulation. Some MSW also expressed fears of infertility and birth defects in their future children if using LA PrEP.

For this sample of men, LA PrEP was seen as potential alternative options to overcome the challenges of condoms and oral PrEP use and provided an acceptable tool to reduce the risk of acquiring HIV. Injectable or implant options offered longer lasting duration and HIV protection and reduced the burden of frequent clinic visits for refills. The dislike for monthly clinic visits because of stigma, long queues, waiting hours, and cost was evident. Findings revealed that both MSW and MSM perceived stigma as a significant concern. Participants expressed fears about how other men in their communities would react to their use of HIV prevention methods. This could potentially compromise the acceptability of the bimonthly injectable PrEP cabotegravir that is now recommended by the World Health Organization as an additional prevention choice for people at substantial risk, including MSM.^32,33^ Interestingly, the sources of stigma differed between the 2 subgroup male populations in this study. MSW expressed concerns about stigma related to public clinic attendance and health seeking, which may have been reflective of their perceived masculinity or societal expectations. A study in South Africa found that men perceived public clinics as places for women and reported negative experiences with female nurses, perceiving them to be rude and judgmental.^34^ In contrast, MSM reported product-related stigma, voicing that they were hesitant to use implants because of its association with being a product for women. MSM exhibited a heightened awareness of health disparities and specific health care needs associated with their sexual orientation. This awareness sensitized them to the potential stigma they might face when seeking health care services. Moreover, there was a fear among some MSM individuals that using products traditionally associated with women could compromise their perception of masculinity within the community. Further exploration of these differences can provide valuable insights into addressing cisgender men’s experiences and fears of stigma in various contexts, such as the establishment of men-specific clinics to normalize health care-seeking behaviors among men in these communities.

Another important concern that emerged was the perception of long-acting HIV prevention methods as “foreign objects.” Men expressed mistrust toward products manufactured outside their country and discomfort with the idea of having a physical object remain inside their bodies, even if the product was locally manufactured. The topic of new prevention methods and mistrust and fear of foreign-made HIV prevention products in African context is salient and not entirely novel.^35^ It sheds light on the historical relationships between the Global North and Global South, where there has been a perception that certain prevention methods are developed with the intent to reduce the African population.^36^ User-centered design approaches, such as “demand creation”^37^ could minimize this mistrust by addressing men’s concerns and providing clear information about the safety and efficacy of the products, and concerns about LA PrEP possibly affecting fertility. The association of sexual and reproductive health prevention methods (eg, vaccines, contraceptives) and fear of infertility is a phenomenon that is common among users in this setting^35,36,38^ and speaks of the need for community-wide education and sensitization. Moreover, although an implant may not be suitable for everyone, offering a range of HIV prevention method choices is valuable, because implants offer the benefit of longer duration and fewer clinic visits. A study in Canada found that some oral PrEP users preferred the convenience, adherence, and confidentiality of injectables, while others feared needles or felt more “in control” with oral PrEP,^39^ illustrating the importance of being offered a choice.

The notion of “foreign objects” also reflects a sense of “othering,” where men perceived the LA products as something external and different from themselves. This feeling of “othering” may stem from a general internalized stigma that associates certain HIV prevention methods with promiscuity or engaging in high-risk behaviors, in turn leading to feelings of shame or inferiority among some men. A study conducted in the United States reported that the fear of being stigmatized for taking PrEP was often rooted in participant’s own stigmatization of people living with HIV. This association carried notions of rejection, isolation, an ostracization, thus the global framing of PrEP being targeted only to certain “high-risk” population groups in clinical guidelines, and the persistent focus on risk in the framing of PrEP, enhances perceptions of PrEP being linked to risky sex, sexual irresponsibility, and promiscuity, which in turn adds another aspect to PrEP-related stigma.^14,40^ Moreover, experiences of external stigma play a role in reinforcing this “othering” phenomenon. Men may have observed or experienced stigma directed toward individuals who use HIV prevention or treatment methods, further solidifying their reluctance to associate themselves with such prevention methods. The issue of “othering” extends beyond the LA products themselves and encompasses the broader context of HIV prevention, where certain prevention methods become linked to other factors including gender and sexual identities.^41^ For instance, a study conducted in South Africa revealed that despite decades of efforts to destigmatize HIV/AIDS, it was still linked to being homosexuality or attributed to women, perpetuating negative perceptions of promiscuity, immorality, and uncleanliness.^42^ The roots of “othering” lie in fear and the unknown, especially when LA products and HIV prevention methods, particularly for MSW, may be unfamiliar or erroneously associated with contexts such as contraception or being homosexual. To counteract this, increasing exposure and experience with these methods within various communities can enhance their acceptability among all men and reduce the impact of “othering” based on misconceptions. By fostering understanding and breaking down stigma, we can promote a more inclusive and empathetic approach to sexual health promotion.

This study also sheds light on the negative perceptions of clinics among men, with many participants expressing strong dislike or even hatred toward the clinic setting where HIV prevention services are provided. This sentiment highlights the need for policy and implementation changes that prioritize user-centered approaches. Establishing new places and spaces specifically designed for men’s health, addressing their unique needs and preferences, could help alleviate their aversion to clinics. A similar study conducted in Canada found that some men were not interested in injectable PrEP because of perceived time-consuming nature of returning to the clinic every 2 months.^39^ Men might have a different conceptualization of time compared with women, which becomes crucial to minimizing the time and frequency spent at clinics as they might often experience impatience and find the structure and context of clinics to be inefficient and frustrating. Specifically, waiting in lines and wasting time are particularly unappealing aspects of the clinic experience for men. Thus, creating male-friendly dispensing options that are convenient and efficient could effectively alleviate their frustrations and potentially overcome the stigma-related barriers. A recent study compared attrition from care between men initiating antiretroviral therapy in male-only clinics and those in public clinics. The findings revealed that men attending male-targeted clinics experienced lower attrition rates than those in public clinics. Furthermore, men initiating care in male-only clinics demonstrated slower HIV progression than their counter-parts in public clinics.^43^

The findings of our study have certain limitations that should be considered. First, it is important to note that the views expressed by participants in this study were based on hypothetical scenarios, because they did not have actual experiences using injectable or implant PrEP, thus findings could have differed had they reported their perspectives based on actual usage. It is also worth mentioning that these participants were willing to use HIV prevention methods and as a result, the findings may not be representative of the broader South African male population. In addition, the research was conducted in a clinical environment, which could have influenced participant’s responses regarding their interest in long-acting HIV prevention methods. Finally, participants are representative of peri-urban and urban dwellers and thus perspectives might differ from men in rural settings. However, despite these limitations, these findings provide important information regarding challenges that could hinder men from accepting new HIV prevention technologies and some insights on tackling these potential obstacles.

## CONCLUSIONS

Evidence relating to men’s engagement in HIV prevention and which modalities of HIV prevention may be acceptable and preferred is limited. We found that both MSW and MSM were enthusiastic about the concept of these 2 delivery options for LA PrEP. The findings of this study emphasize the importance of user-centered design in addressing men’s concerns and preferences regarding HIV prevention tools. Discretion, stigma reduction, addressing mistrust, offering choices, and creating male-friendly health care settings are key considerations in the development and implementation of effective HIV prevention strategies for men. By prioritizing these factors, it is possible to improve the acceptability and effectiveness of HIV prevention methods among men, ultimately contributing to the reduction of HIV transmission rates. This research was used to inform the development of a clinical study to provide further insight into safety, acceptability, and use of placebo versions of LA PrEP among MSW and MSM.

## Figures and Tables

**TABLE 1.** Demographic Characteristics of Participants in the Qualitative Study (N = 40)

	n (%) or Median (Min–Max)

Age	23.5 (18–38)
Ethnic group	
Zulu	6 (15.0)
Xhosa	22 (55.0)
Sotho	6 (15.0)
Colored	1 (2.5)
Other African tribe	5 (12.5)
Language most spoken at home	
IsiZulu	7 (17.5)
IsiXhosa	21 (52.5)
English	4 (10.0)
Sesotho	3 (7.5)
Other	5 (12.5)
Highest level of education	
Primary school, not complete	1 (2.5)
Secondary school, not complete	5 (12.5)
Secondary school, complete	20 (50.0)
Attended college or university, not complete	11 (27.5)
Attended college or university, complete	3 (7.5)
Currently in school	13 (32.5)
Sexual identity	
Gay	17 (42.6)
Straight	19 (47.5)
Bisexual	4 (10.0)
Has a primary partner	32 (80.0)
Condom used at last sex	19 (47.5)
Source of income	
None	30 (75.0)
Formal employment	5 (12.5)
Self-employment	2 (5.0)
Social grant	3 (7.5)
Trial experienced	
MSW	1 (2.5)
MSM	12 (30.0)
Trial naive	
MSW	19 (47.5)
MSM	8 (20.0)
